# Emerging CAM *Ziziphus nummularia* with in vivo sedative-hypnotic, antipyretic and analgesic attributes

**DOI:** 10.1007/s13205-015-0322-5

**Published:** 2016-01-05

**Authors:** Abdur Rauf, Jawad Ali, Haroon Khan, Mohammad S. Mubarak, Seema Patel

**Affiliations:** 1 Department of Geology, University of Swabi, Anbar, Khyber Pakhtunkhwa 23561 Pakistan; 2 Institute of Chemical Sciences, University of Peshawar, Peshawar, KP 25120 Pakistan; 3 Department of Pharmacy, Abdul Wali Khan University, Mardan, 23200 Pakistan; 4 Department of Chemistry, The University of Jordan, Amman, 11942 Jordan; 5 Bioinformatics and Medical Informatics Research Center, San Diego State University, San Diego, CA 92182 USA

**Keywords:** *Ziziphus nummularia*, Sedative, Hypnotic, Antipyretic, Antinociceptive, Anti-inflammation

## Abstract

*Ziziphus nummularia* from Rhamnaceae family is traditionally used for sedative-hypnotic, antipyretic and analgesic purposes; however, scientific validations are lacking. This in vivo study was undertaken to verify the above ameliorative properties of *Z*. *nummularia* root methanolic extract. Various fractions of the extract were assayed on Balb/c mice by open field, Brewer’s yeast-induced hyperthermia and acetic acid-induced writhing experiments. The significance of the outcomes was analyzed with statistical tests. Various fractions of the extract exhibited marked dose-dependent (*p* < 0.05) sedative-hypnotic and antipyretic activities. The biological efficacies were most pronounced between 50 and 100 mg/kg. Further, the acetic acid-induced abdominal constrictions were significantly (*p* < 0.05) attenuated by the extract. Chloroform fraction of the extract was most dominant followed by ethyl acetate. The demonstrated therapeutic attributes of *Z*. *nummularia* extract can be exploited to isolate pharmaceutically relevant compounds.

## Introduction

Since the dawn of human race, plants are being used as medicines (Petrovska 2012). World Health Organization (WHO) statistics reflects that 80 % of the world population, mostly from developing countries still rely on botanical for health care (Khan 2014). A vast majority of conventional drugs and most of the complementary and alternative medicines (CAM) are plant-derived (Mainardi et al. 2009). For their benign toxicity compared to chemotherapeutics, phytochemicals are widely used against a plethora of infectious, metabolic and degenerative diseases (Pandey and Rizvi 2009). The health restorative applications of plant products are exhaustive, yet the significant are antioxidant, anti-inflammatory, anticancer, anti-diabetic, analgesic, antimicrobial, antiulcer, etc. (Benzie and Wachtel-Galor 2011). The key phytochemicals have been characterized to be alkaloids, phenolic acids, flavonoids, terpenoids, sterols, saponins, tannins, etc., though each species has its distinct botanical repertoire. Most plants are geographically confined and the medicinal uses largely hinge on their local distribution and traditional knowledge on them (Saslis-Lagoudakis et al. 2014).

In this resource-depleted world, sustainability requires the search for food and medicine in the vicinity (Cordell 2011). *Ziziphus* is a genus in family Rhamnaceae with global distribution (Zhao et al. 2014). The common species of this genus are *Z. jujube*, *Z. zizyphus*, *Z. mauritiana*, *Z. joazeiro*, *Z. lotus*, *Z. spina*-*christi*, Z, *spinosa*, *Z. oxyphylla*, *Z. oenoplia* and *Z. oxyphylla* Edgew (Pawlowska et al. 2009). The ethnobotanical, phytochemical, economical, and pharmacological attributes of many species have been extensively studied. Some popular food and medicinal uses of this genus have been mentioned below. The drupes ‘jujube or Chinese dates’ are nutritious and popular fruits (Gao et al. 2013b). They are often processed into pulps, polysaccharides, antioxidants, pickles, jam, fermented (masau) (Adeli and Samavati 2014; Li et al. 2014; Elaloui et al. 2015). Usage of this genus as dietary supplements and herbal medicines in Tunisia (Elaloui et al. 2015), China (Plastina et al. 2012), Japan (Oshima et al. 2015), Korea (Kim et al. 2013), and other Asian countries (San et al. 2013) have been well-documented (Chen et al. 2014b). Many of the folkloric practices have been empirically validated now. The verified health benefits include antioxidant (Olajuyigbe and Afolayan 2011; Chen et al. 2014c), anti-inflammation (Yu et al. 2012), antibacterial and antifungal (Ghazghazi et al. 2014), antiulcerogenic (Wahida et al. 2007), immunomodulatory (Chen et al. 2014a), diabetes ameliorating (Goli-malekabadi et al. 2014), hepatoprotective (Kwape et al. 2013; Yue et al. 2014), hematopoietic (Chen et al. 2014b), antimutagenic (Boriollo et al. 2014), cytotoxic (Jafarian et al. 2014), and sedative-hypnotic (Gao et al. 2013a). The plant parts conferring the biological effects are diverse such as leaves (Nisar et al. 2007; Kwape et al. 2013; Goyal et al. 2013), fruits (Plastina et al. 2012; Chen et al. 2014a, b, c), seeds (Cao et al. 2010; San et al. 2013; Gao et al. 2013a), bark (Olajuyigbe and Afolayan 2011; Boriollo et al. 2014) and root (Rao et al. 2012).

**Fig. 1:**
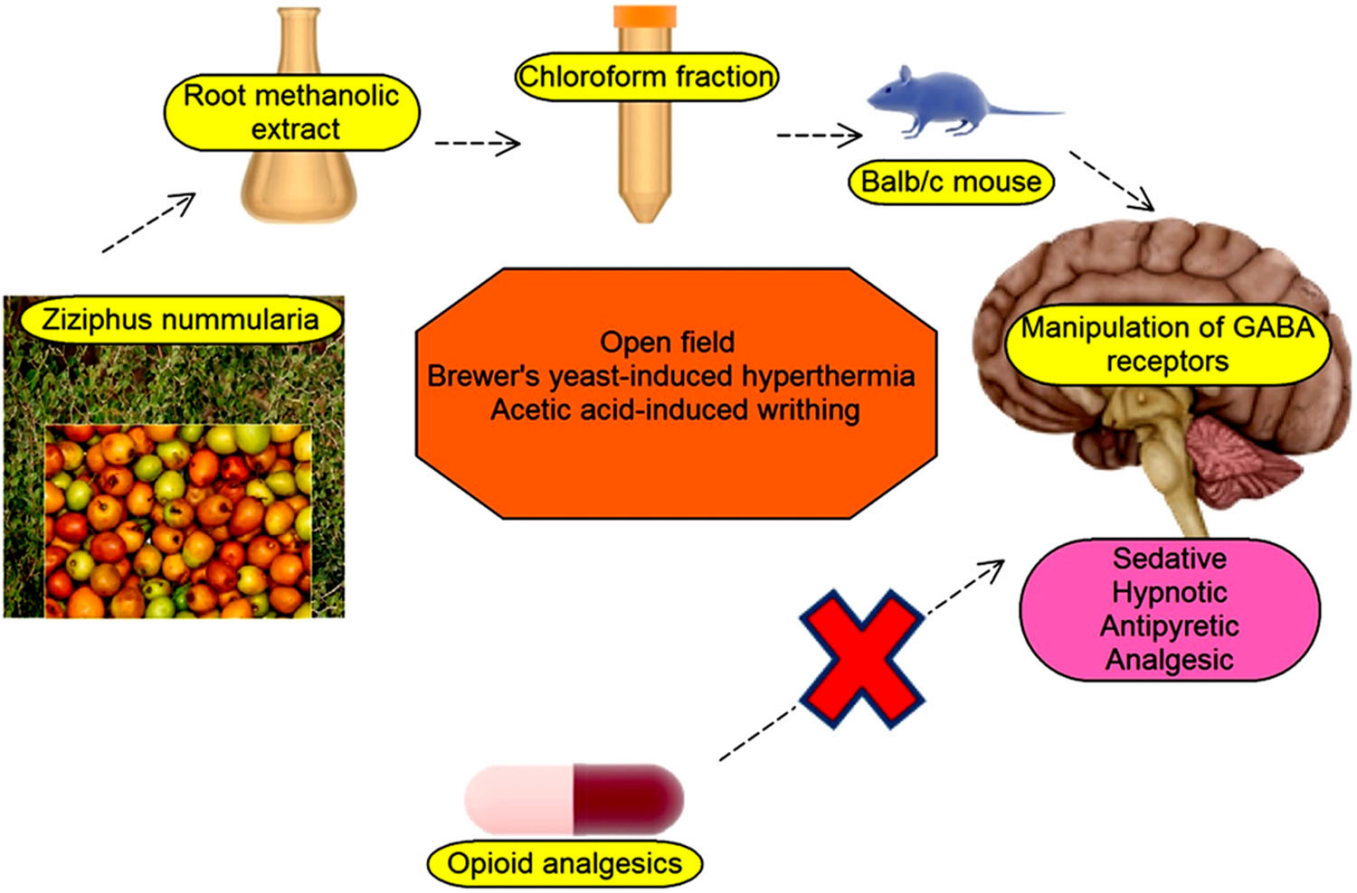
The overview of *Z. nummularia* extract eliciting sedative, hypnotic, antipyretic, analgesics effect in balb/c mice

*Ziziphus nummularia*, is a species of the above genus, and native to the arid regions of India (Bachaya et al. 2009), Pakistan (Abbasi et al. 2013), Afghanistan, Iraq, Iran, Egypt, and Israel (Desai et al. 2008). The versatile uses of this species have been depicted in an insightful review (Pandey et al. 2010). Ethnobotanical survey for wild edible and medicinal plants have projected it as a major species in tribal communities of Lesser Himalayas-Pakistan (Abbasi et al. 2013). This plant is known for its palatable and vitamic C-rich drupes. Literature search reflects its usage as an astringent, treatment of scabies and other skin diseases (Khare 2003). Its use as antidiarrheal in Sariska region of India (Upadhyay et al. 2011) and anthelmintic in Pakistan (Bachaya et al. 2009) is prevalent. Also, the analgesic and anti-inflammatory efficacy of its cyclopeptide alkaloid-rich leaf extract has been reported (Goyal et al. 2013). However, there is no information of its beneficial role in insomnia, anxiety, pain, and fever. Though, some other species of Ziziphus genus have been proven effective in easing those ailments such as *Z. jujube* (Jiang et al. 2007; Cao et al. 2010; Yeung et al. 2012; Awad et al. 2014), *Z. oxyphylla* Edgew (Nisar et al. 2007; Kaleem et al. 2013), *Z. mauritiana* (San et al. 2013). So, this in vivo study was undertaken to evaluate the sedative-hypnotic, antipyretic and analgesic activities of *Z*. *nummularia* root extracts as a pharmacological justification of its traditional usage and premise for future drug discovery (Fig. 1).

## Materials and methods

### Plant material

The roots of *Z. nummularia* were collected from Takht Bhai District Mardan, Khyber Pakhtunkhwa, Pakistan. The plant was identified and authenticated by Professor Dr. Abdur Rashid, a botanist at the Botany Department, University of Peshawar, Pakistan. A voucher specimen (BOT 200399) has been deposited in the herbarium of the department of Botany, University of Peshawar, Pakistan.

### Experimental animals

Balb/c mice of both gender, each weighing about 24–26 g were kept under standard laboratory conditions at (25 ± 4 °C). The animals were fed with a laboratory diet and water ad libitum. All experiments were performed at the Pakistan Council of Scientific and Industrial Research (PCSIR), University of Peshawar, Pakistan and were approved by the local Ethical Committee of the Peshawar University, Pakistan.

### Extraction and fractionation

The root fragments of *Z. nummularia* were shade-dried at room temperature followed by coarse grinding. The powdered material (300 g) was exhaustively extracted with 99 % methanol for 7 days. The extract was concentrated and dried in a rotary evaporator and which yielded 38 g of brownish mass. The solid mass was suspended in water and successively fractionated with various solvents of increasing polarity, and the corresponding fractions of *n*-hexane, chloroform, ethyl acetate, and ethanol were obtained.

### Sedative activity (open field test)

The sedative effect of the crude extract was evaluated according to published procedures using the open field method (Walsh and Cummins 1976). This method has been proven effective in the assessment of locomotor activity and anxiety-linked behaviors (Lau et al. 2008). The apparatus used for the test consisted of a white wooden area (150 cm in diameter) enclosed in stainless steel walls and the area was divided by black lines, into four squares. The open field was placed inside a light and sound-attenuated room. The mice were adapted to red light (40 W) for 60 min prior to the start of experiment and fed ad libitum. The animals were administered with normal saline and *Z. nummularia* root extract at the doses of 50 and 100 mg/kg. After 30 min of the treatment, each mouse was placed in the center of the box and the numbers of lines crossed were counted.

### Hypnotic properties in phenobarbitone-induced sleeping time

The test mice were divided into groups of six each. Mice of the control group (negative control) were orally treated with normal saline (10 ml/kg) whereas those of the positive control group were given the standard drug bromazepam (5 mg/kg). This drug, from the class benzodiazepines (BZD) imparts short-term relief from anxiety by modulating the neurotransmitters in brain (Mandrioli et al. 2010). The remaining mice were treated with the *Z. nummularia* root extract at 50 and 100 mg/kg doses. After 30 min of treatment, all animals were injected with the barbiturate drug phenobarbitone sodium at a dose of 35 mg/kg and observed for onset and duration of sleep. The hypnotic effect of the extract was evaluated.

### Brewer’s-yeast-induced hyperthermia

The antipyretic activities of *Z. nummularia* root extract were assessed in Brewer’s yeast (*Saccharomyces cerevisiae*)-induced hyperthermia (Tomazetti et al. 2005). Pyrexia was induced in mice by injection of 10 ml/kg of 15 % suspension of yeast. The normal body temperature of the animals was recorded with a digital clinical thermometer (Hartmann, Germany) using rectum, while the tail was fastened with an adhesive tape. The rectal temperature of each mouse was measured again after 19 h of yeast injection as described above. Mice that developed a minimum increase of 0.5 °C or more temperature were selected for the experiment. These rodents were categorized in group of size 6. A group of mice was fed with saline (10 ml/kg) and used as negative control, whereas other groups was treated either with paracetmol (100 mg/kg through intraperitoneal injection) as standard drug or *Z. nummularia* root extract at the doses of 50 and 100 mg/kg. After the respective treatment, the rectal temperature of each animal was again recorded at 1 h intervals for following 5 h. The obtained data were used for the calculation of percentage reduction in rectal temperature. Antipyretic activity was defined as the efficacy of test drug to abate the induced-pyrexia (Muhammad et al. 2013).

$${\text{Reduction in temperature }}\left( \% \right) = \frac{B - C}{B - A} \times 100$$

where* A* is the normal body temperature,* B* is the temperature after pyrexia induction,* C* is the temperature after 1, 3 and 5 h.

### Acetic acid-induced writhing test

Intraperitoneal injection of acetic acid triggers writhing, most likely due to the release of TNF-α, interleukin 1β and interleukin 8 by macrophages and mast cells (Ribeiro et al. 2000). The mice were divided into groups of 6 for evaluation of the analgesic effect of *Z. nummularia* roots extract. One group was injected with normal saline (10 ml/kg) and used as negative control. Another group was injected with diclofenac (5 and 10 mg/kg). The last group was treated with *Z. nummularia* root extract at 50 and 100 mg/kg. The mice were injected with acetic acid (1 % intraperitoneal) and the subsequent abdominal constriction (writhing) was counted for 10 min after 5 min of acetic acid injection.

### Statistical analyses

The results are expressed as the mean ± standard error of the mean (SEM). One-way analysis of variance (ANOVA) was used for analysis of data followed by Dunnett’s test for multiple comparisons (Hoffman et al. 2008). Differences were considered significant at *p* ≤ 0.05.

## Results and discussion

### Effect of extract/fractions in open field test

**Fig. 2:**
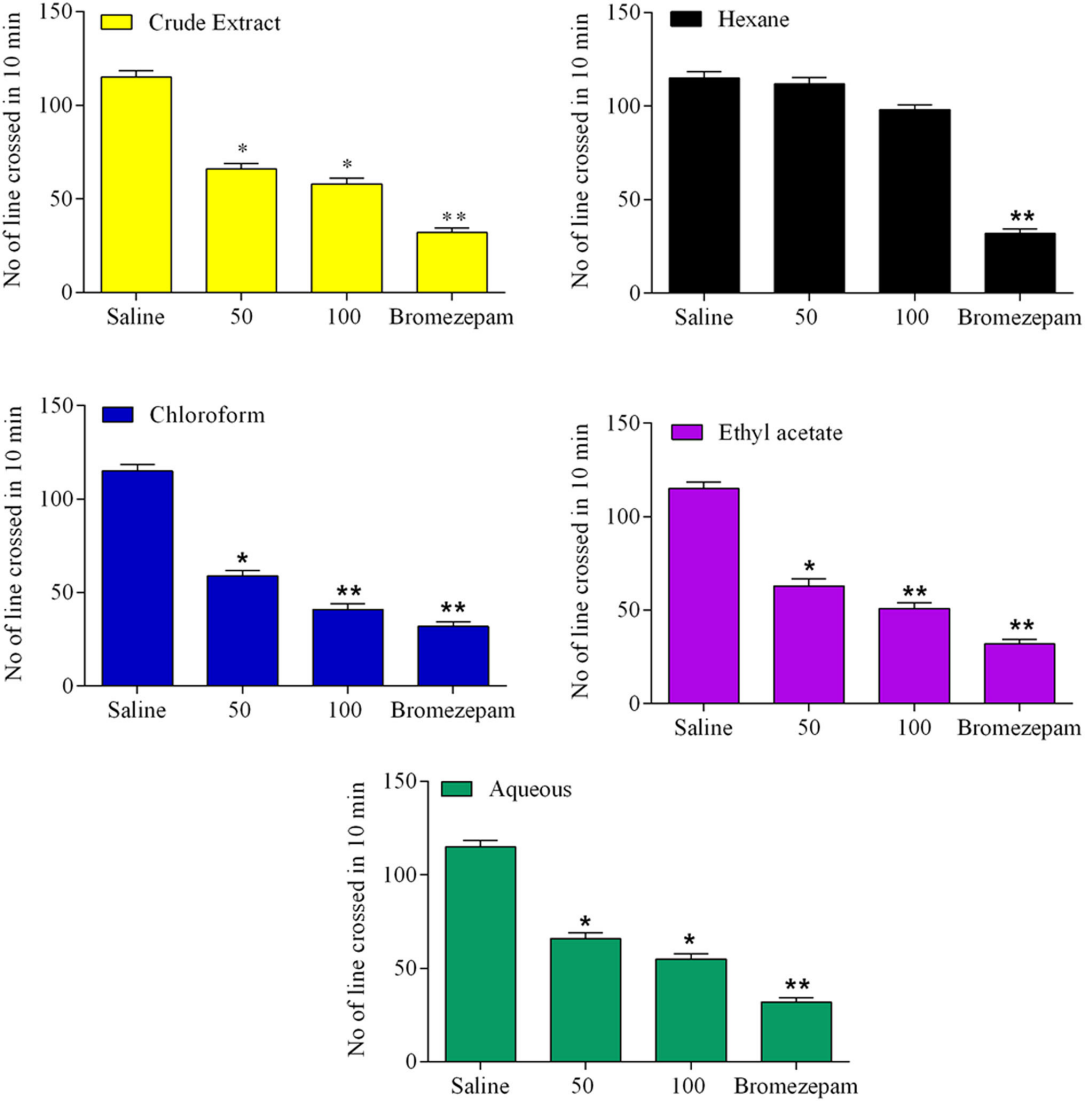
Data shows the number of lines crossed by animals in a *box*, 30 min after treatment with normal saline (10 ml/kg, control), extract/fractions of *Z. nummularia* roots (50 and 100 mg/kg) or bromazepam (5 mg/kg). Values are given as mean ± S.E.M, (*n* = 6). *p < 0.05 or **p < 0.01, *p* < 0.001, all compared with control

The efficacy of intraperitoneally administered *Z. nummularia* root extract (50 and 100 mg/kg) and bromazepam in mice have been illustrated in Fig. 2. The extract showed marked sedative effect with decreased locomotion in dose-dependent manner. The chloroform fraction was the most dominant followed by ethyl acetate with (*p* < 0.05 and *p* < 0.01) at 50 and 100 mg/kg.

### Effect of extract in open field test

The effect of *Z. nummularia* roots extract at 50 and 100 mg/kg, in phenobarbitone-sleep in mice is shown in Table 1. The extract significantly (*p* < 0.05) reduced the sleep latency time (time taken for the onset of sleep) and increased the sleep in dose-dependent manner. Chloroform followed by ethyl acetate extract was the most potent fraction in inducing sleep and increasing its duration. The soporific effect elicited by the extract in bromazepam treated group was highly significant (*p* < 0.001).

**Table 1:** The effect of *Z. nummularia* root fractions in phenobarbitone-induced sleep in mice

Drug	Intraperitoneal dose	Onset of sleep (min)	Duration of sleep (min)
Normal saline	10 ml/kg + phenobarbitone	23.27 ± 1.09	7.56 ± 2.45
Crude extract	50 mg/kg + phenobarbitone	18.10 ± 2.70	15.40 ± 1.75
	100 mg/kg + phenobarbitone	14.33 ± 1.08*	23.88 ± 2.75*
Hexane	50 mg/kg + phenobarbitone	19.66 ± 1.12	9.45 ± 0.67
	100 mg/kg + phenobarbitone	19.00 ± 1.05	9.60 ± 1.06
Chloroform	50 mg/kg + phenobarbitone	15.33 ± 1.21*	22.33 ± 1.12*
	100 mg/kg + phenobarbitone	10.56 ± 0.99**	27.05 ± 1.35**
Ethyl acetate	50 mg/kg + phenobarbitone	18.44 ± 1.12*	20.12 ± 1.45*
	100 mg/kg + phenobarbitone	12.57 ± 1.49*	23.67 ± 1.56*
Aqueous	50 mg/kg + phenobarbitone	17.44 ± 1.06	17.04 ± 0.99*
	100 mg/kg + phenobarbitone	13.03 ± 0.89*	23.55 ± 1.77*
Bromazepam	5 mg/kg + phenobarbitone	3.25 ± 1.10***	61.45 ± 1.10***

* Data shows the onset and duration of sleep (in min) in mice pretreated with normal saline (10 ml/kg), extract/fractions (50 and 100 mg/kg) or bromazepam (5 mg/kg) and then all groups were treated with phenobarbitone (35 mg/kg). Data presented as mean ± S.E.M, (*n* = 6). * *p* < 0.05, ** *p* < 0.01, *** *p* < 0.001, all with respect to control

### Effect of extract in yeast induced hyperthermia

**Fig. 3:**
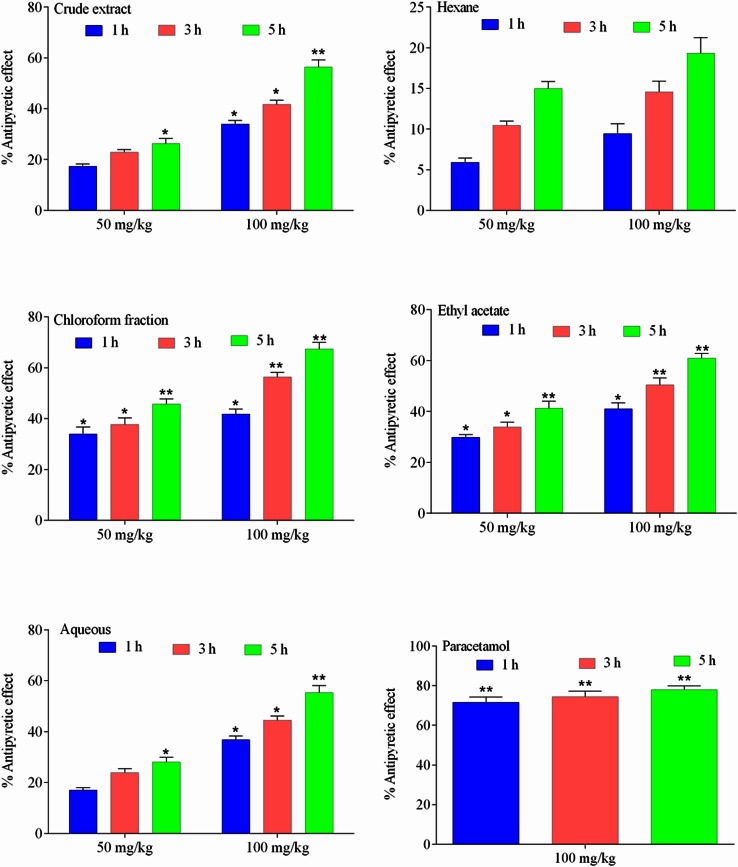
The percent antipyretic effect of extract/fractions of *Z. nummularia* roots in acetic acid-induced writhing. Data presented as mean ± S.E.M, (*n* = 6). *p < 0.05, **p < 0.01, all with respect to control

The antipyretic effect of *Z. nummularia* root extract in yeast-induced hyperthermia has been presented in Fig. 3. The extract produced profound antipyretic effect in a dose-dependent manner during various assessment times (1–5 h). Additionally, the post chloroform treatment caused pronounced reduction in induced pyrexia with 45.77 and 67.44 % effect at 50 and 100 mg/kg, respectively after 1 h of injection.

### Effect of extract in acetic acid induced-writhing

**Fig. 4:**
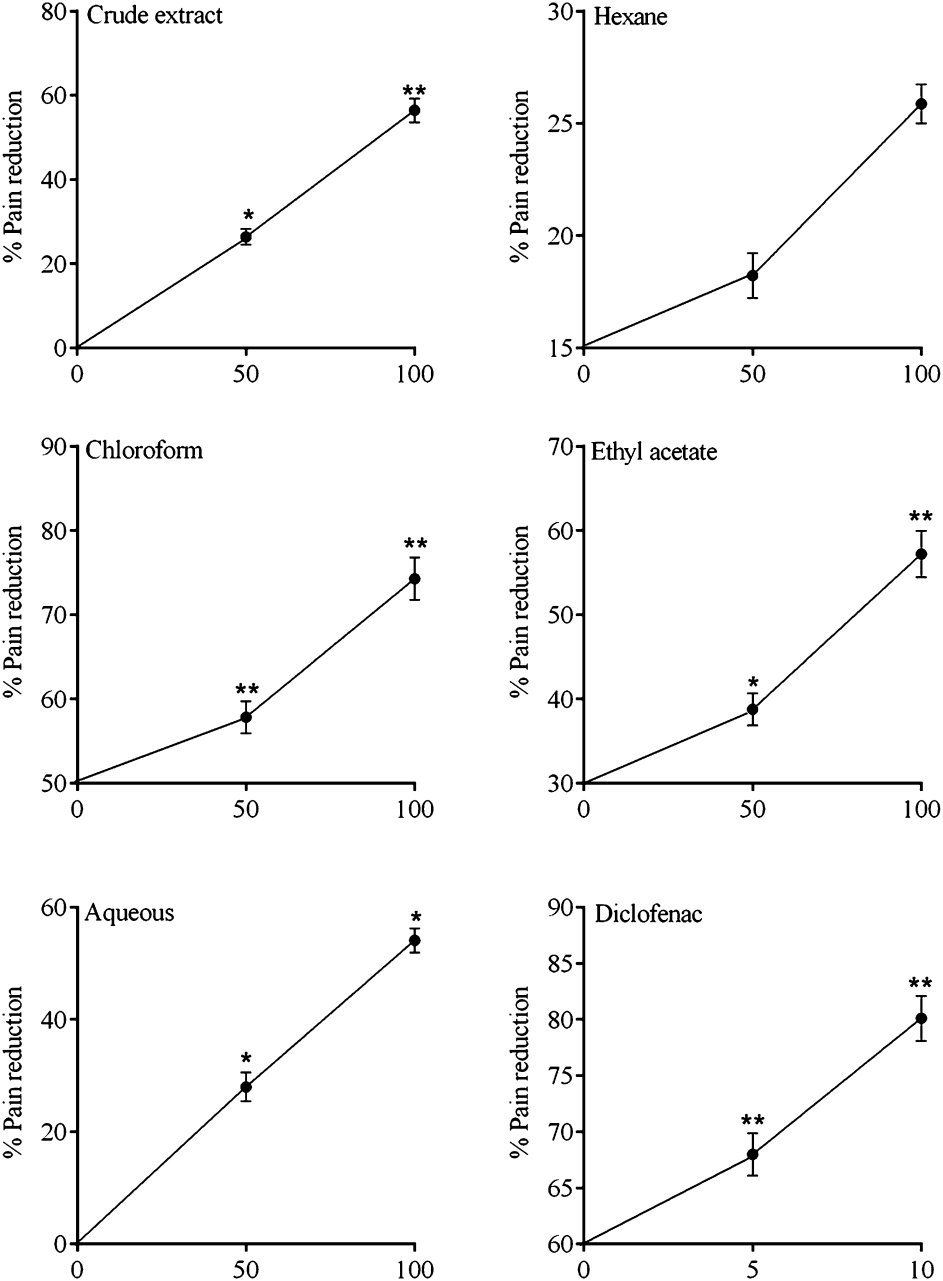
The percent antinociceptive effect of extract/fractions of *Z. nummularia* roots in acetic acid-induced writhing. Data presented as mean ± S.E.M, (*n* = 6). *p < 0.05, **p < 0.01, ***p < 0.001, all with respect to control

The antinociceptive activity of the *Z. nummularia* root extract has been shown in Fig. 4. As per the result, the extract significantly reduced pain sensation in acetic acid-induced writhing test. For all fractions of the extract, dose-dependent effects have been observed at 50 and 100 mg/kg, and the analgesic effect of chloroform fraction was most pronounced with 57.86 and 74.28 % at 50 and 100 mg/kg, respectively.

## Discussion

The findings from this investigation suggest that the roots extract of *Z. nummularia* displays significant in vivo sedative-hypnotic, antipyretic, and analgesic effects. The open field test is one of the commonly used behavioral test protocol for screening of sedative and anxiolytic activity of substances in laboratory animals (de la Peña et al. 2013), whereas the phenobarbitone-induced sleeping time test (Inganakal et al. 2012) is employed for the assessment of hypnotic properties. The roots extract significantly reduced the movement of mice in the open field test apparatus implying the tranquilizing nature of its components. While the precise mechanistic of therapeutic role is not known, several probable causes were formulated. The allosteric modulation of γ-aminobutyric acid (GABA) receptors in mice brain might be the underlying mechanism (Shrestha et al. 2012). The prolongation of the sleeping duration suggested the hypnotic properties of the extract. The effect could have been mediated by the induction of central nervous system (CNS) depressant activity by the bioactive components of the extract (Ya’u et al. 2011). In this investigation, the crude extracts showed remarkable sedative-hypnotic effect which varied upon fractionation. The chloroform fraction was most potent followed by ethyl acetate whereas the hexane fraction did not produce significant results. From the results, it could be assumed that the sedative-hypnotic constituents of *Z. nummularia* roots are mostly concentrated in chloroform and ethyl acetate fractions. Results revealed that *Z. nummularia* root extract elicits antipyretic effects in yeast-induced hyperthermia in the murine models. Chloroform fractions proved to be the most active fraction followed by ethyl acetate, suggesting the presence of pharmacologically important components in them.

The acetic acid-induced writhing test is considered very effective in detecting antinociceptive effects of various extracts. Its sensitivity is much higher than other methods (hot plate, tail flick, carrageenan-induced paw edema, formalin-induced paw licking, etc.) used for this purpose (Ramirez et al. 2010). The abdominal constrictions and the resultant painful sensations provoked by the acid has been attributed to the perturbation and interplay of bradykinin, prostaglandins, interleukin-1β (IL-1β), interleukin-8 (IL-8), and tumor necrosis factor-α (TNF-α) (Ribeiro et al. 2000). The *Z. nummularia* root extract at the doses of 50–100 mg/kg, moderated the abdominal constrictions in a dose-dependent manner. One possible mechanism of this action could be the diminished production of prostaglandin E2 (PGE2) (Ying et al. 2014). The extract could be interfering with both central (opioid and cholinergic pathways) and peripheral (COX-2) pathways in eliciting the anti-inflammatory effects (Sawada et al. 2014). The chloroform fraction was the most effective followed by the ethyl acetate fraction in reducing pain and sensitivity. It indicates the accumulation of the antinociceptive constituents in these fractions.

Anxiety and sleep interruptions have become the norm of current stressful living (Han et al. 2012). Also, the instances of depression, suicidal attempts are soaring (Cash and Bridge 2009). Reliance on the prescription drugs is not free of side effects. The frequent intake aggregates the metabolic system in long term, becomes ineffective and overdose proves fatal. Opioid analgesics (buprenorphine, fentanyl, codeine, ativan, dextropropoxyphene, methadone, oxycodone, carisoprodol, etc.) overdose-caused death cases are rising dramatically (Paulozzi et al. 2012). The deadly combination opioid, benzodiazepines, and/or alcohol use poses huge risk of morbidity (Gudin et al. 2013). On the other hand, CAM are naturally sourced, thus pose lower genotoxicity compared to chemicals. In this regard, *Z. nummularia* root extract merits further investigation. Isolation of the functional constituents and their structural elucidation warrants impetus. In a preliminary screening study, the phytochemicals alkaloids (ziziphines), carbohydrates, saponins, flavonoids, anthocyanins, and tannins were found in the ethanolic extract of *Z*. *oenoplia* (Suksamrarn et al. 2005; Rao et al. 2012). These components might be instrumental in inducing the tested biological responses. The hypnotic efficacy of *Z. jujuba* Mill. jujubosides has been correlated to the impact on circadian rhythm and the serotonergic system (Cao et al. 2010). Further studies on the therapeutic importance of this plant might provide affordable medication to large section population in the developing countries. However, possible health risks must be verified for broader remedial applications.

## Conclusion

The cumulative findings from this study suggest that the root extract of *Z. nummularia* exhibits remarkable sedative-hypnotic, antipyretic and analgesic effects in mice. These findings explain the rationale of traditional medicinal use of *Z. nummularia* for alleviating pain, fever, insomnia, seizures, muscle spasms, anxiety etc. However, more in-depth studies are required to establish the safety, efficacy, and active constituents of this plant. This study is expected to inspire further investigation into this underutilized medicinal plant.

## References

[CR1] Abbasi AM, Khan MA, Khan N, Shah MH (2013). Ethnobotanical survey of medicinally important wild edible fruits species used by tribal communities of Lesser Himalayas-Pakistan. J Ethnopharmacol.

[CR2] Adeli M, Samavati V (2014). Studies on the steady shear flow behavior and chemical properties of water-soluble polysaccharide from *Ziziphus lotus* fruit. Int J Biol Macromol.

[CR3] Awad DS, Ali RM, Mhaidat NM, Shotar AM (2014). *Zizyphus jujuba* protects against ibuprofen-induced nephrotoxicity in rats. Pharm Biol.

[CR4] Bachaya HA, Iqbal Z, Khan MN (2009). Anthelmintic activity of *Ziziphus nummularia* (bark) and Acacia nilotica (fruit) against Trichostrongylid nematodes of sheep. J Ethnopharmacol.

[CR5] Benzie IFF, Wachtel-Galor S (2011) Herbal medicine 2nd edition, Biomolecular and Clinical Aspects, Oxidative Stress and Disease. ISBN-13: 978-1-4398-0713-2

[CR6] Boriollo MFG, Resende MR, da Silva TA (2014). Evaluation of the mutagenicity and antimutagenicity of *Ziziphus joazeiro* Mart. Bark in the micronucleus assay. Genet Mol Biol.

[CR7] Cao J-X, Zhang Q-Y, Cui S-Y (2010). Hypnotic effect of jujubosides from Semen Ziziphi Spinosae. J Ethnopharmacol.

[CR8] Cash SJ, Bridge JA (2009). Epidemiology of youth suicide and suicidal behavior. Curr Opin Pediatr.

[CR9] Chen J, Du CYQ, Lam KYC (2014). The standardized extract of *Ziziphus jujuba* fruit (jujube) regulates pro-inflammatory cytokine expression in cultured murine macrophages: suppression of lipopolysaccharide-stimulated NF-κB activity. Phytother Res.

[CR10] Chen J, Lam CTW, Kong AYY (2014). The extract of *Ziziphus jujuba* fruit (Jujube) induces expression of erythropoietin via hypoxia-inducible factor-1α in cultured Hep3B cells. Plant Med.

[CR11] Chen J, Yan AL, Lam KYC (2014). A chemically standardized extract of *Ziziphus jujuba* fruit (jujube) stimulates expressions of neurotrophic factors and antioxidant enzymes in cultured astrocytes. Phytother Res.

[CR12] Cordell GA (2011). Sustainable medicines and global health care. Plant Med.

[CR13] De la Peña JBI, Lee HL, Yoon SY (2013). The involvement of magnoflorine in the sedative and anxiolytic effects of Sinomeni Caulis et Rhizoma in mice. J Nat Med.

[CR14] Desai AG, Qazi GN, Ganju RK (2008). Medicinal plants and cancer chemoprevention. Curr Drug Metab.

[CR15] Elaloui M, Laamouri A, Fabre J (2015). Distribution of free amino acids, polyphenols and sugars of *Ziziphus jujuba* pulps harvested from plants grown in Tunisia. Nat Prod Res.

[CR16] Gao J-R, Ji W-B, Jiang H, Chen J-F (2013). Effects of extracts from ziziphi spinosae semen and schisandrae chinensis fructus on amino acid neurotransmitter in rats with insomnia induced by PCPA. Zhong Yao Cai.

[CR17] Gao Q-H, Wu C-S, Wang M (2013). The jujube (*Ziziphus jujuba* Mill.) fruit: a review of current knowledge of fruit composition and health benefits. J Agric Food Chem.

[CR18] Ghazghazi H, Aouadhi C, Riahi L (2014). Fatty acids composition of Tunisian *Ziziphus lotus* L. (Desf.) fruits and variation in biological activities between leaf and fruit extracts. Nat Prod Res.

[CR19] Goli-malekabadi N, Asgary S, Rashidi B (2014). The protective effects of *Ziziphus vulgaris* L. fruits on biochemical and histological abnormalities induced by diabetes in rats. J Complement Integr Med.

[CR20] Goyal M, Ghosh M, Nagori BP, Sasmal D (2013). Analgesic and anti-inflammatory studies of cyclopeptide alkaloid fraction of leaves of *Ziziyphus nummularia*. Saudi J Biol Sci.

[CR21] Gudin JA, Mogali S, Jones JD, Comer SD (2013). Risks, management, and monitoring of combination opioid, benzodiazepines, and/or alcohol use. Postgrad Med.

[CR22] Han KS, Kim L, Shim I (2012). Stress and sleep disorder. Exp Neurobiol.

[CR23] Hoffman WP, Recknor J, Lee C (2008). Overall type I error rate and power of multiple Dunnett’s tests on rodent body weights in toxicology studies. J Biopharm Stat.

[CR24] Inganakal TS, Ahmed ML, Swamy P (2012). Neuropharmcological potential of methanolic extract and a triterpene isolated from *Madhuca longifolia* L leaves in mice. Indian J Exp Biol.

[CR25] Jafarian A, Zolfaghari B, Shirani K (2014). Cytotoxicity of different extracts of arial parts of *Ziziphus spina*-*christi* on Hela and MDA-MB-468 tumor cells. Adv Biomed Res.

[CR26] Jiang J-G, Huang X-J, Chen J (2007). Separation and purification of saponins from Semen Ziziphus jujuba and their sedative and hypnotic effects. J Pharm Pharmacol.

[CR27] Kaleem WA, Muhammad N, Qayum M (2013). Antinociceptive activity of cyclopeptide alkaloids isolated from Ziziphus oxyphylla Edgew (Rhamnaceae). Fitoterapia.

[CR28] Khan H (2014). Medicinal plants in light of history: recognized therapeutic modality. J Evid Based Complement Altern Med.

[CR29] Khare CP (2003). Indian herbal remedies: rational western therapy, ayurvedic, and other traditional usage, Botany.

[CR30] Kim J-E, Kim M-A, Kim J-S (2013). Enhancing the organoleptic and functional properties of jujube by a quick aging process. Prev Nutr Food Sci.

[CR31] Kwape TE, Chaturvedi P, Kamau JM, George S (2013). Hepato-protective potential of methanol extract of leaf of *Ziziphus mucronata* (ZMLM) against dimethoate toxicity: biochemical and histological approach. Ghana Med J.

[CR32] Lau AA, Crawley AC, Hopwood JJ, Hemsley KM (2008). Open field locomotor activity and anxiety-related behaviors in mucopolysaccharidosis type IIIA mice. Behav Brain Res.

[CR33] Li M, Wang Y, Tsoi B (2014). Indoleacetic acid derivatives from the seeds of *Ziziphus jujuba* var. spinosa. Fitoterapia.

[CR34] Mainardi T, Kapoor S, Bielory L (2009). Complementary and alternative medicine: herbs, phytochemicals and vitamins and their immunologic effects. J Allergy Clin Immunol.

[CR35] Mandrioli R, Mercolini L, Raggi MA (2010). Metabolism of benzodiazepine and non-benzodiazepine anxiolytic-hypnotic drugs: an analytical point of view. Curr Drug Metab.

[CR36] Muhammad N, Saeed M, Khan H (2013). Antipyretic and anticonvulsant activity of n-hexane fraction of *Viola betonicifolia*. Asian Pac J Trop Biomed.

[CR37] Nisar M, Adzu B, Inamullah K (2007). Antinociceptive and antipyretic activities of the *Zizyphus oxyphylla* Edgew. leaves. Phytother Res.

[CR38] Olajuyigbe OO, Afolayan AJ (2011). Phenolic content and antioxidant property of the bark extracts of *Ziziphus mucronata* Willd. subsp. mucronata Willd. BMC Complement Altern Med.

[CR39] Oshima N, Zaima K, Kamakura H (2015). Identification of marker compounds for Japanese pharmacopoeia non-conforming jujube seeds from Myanmar. J Nat Med.

[CR40] Pandey A, Singh R, Radhamani J, Bhandari DC (2010). Exploring the potential of *Ziziphus nummularia* (Burm. f.) Wight et Arn. from drier regions of India. Genet Res Crop Evol.

[CR41] Pandey KB, Rizvi SI (2009). Plant polyphenols as dietary antioxidants in human health and disease. Oxid Med Cell Longev.

[CR42] Paulozzi LJ, Kilbourne EM, Shah NG (2012). A history of being prescribed controlled substances and risk of drug overdose death. Pain Med.

[CR43] Pawlowska AM, Camangi F, Bader A, Braca A (2009). Flavonoids of *Zizyphus jujuba* L. and *Zizyphus spina*-*christi* (L.) Willd (Rhamnaceae) fruits. Food Chem.

[CR44] Petrovska BB (2012). Historical review of medicinal plants’ usage. Pharmacogn Rev.

[CR45] Plastina P, Bonofiglio D, Vizza D (2012). Identification of bioactive constituents of *Ziziphus jujube* fruit extracts exerting antiproliferative and apoptotic effects in human breast cancer cells. J Ethnopharmacol.

[CR46] Ramirez MR, Guterres L, Dickel OE (2010). Preliminary studies on the antinociceptive activity of *Vaccinium ashei* berry in experimental animal models. J Med Food.

[CR47] Rao CV, Rawat AKS, Singh AP (2012). Hepatoprotective potential of ethanolic extract of *Ziziphus oenoplia* (L.) Mill roots against antitubercular drugs induced hepatotoxicity in experimental models. Asian Pac J Trop Med.

[CR48] Ribeiro RA, Vale ML, Thomazzi SM (2000). Involvement of resident macrophages and mast cells in the writhing nociceptive response induced by zymosan and acetic acid in mice. Eur J Pharmacol.

[CR49] San AMM, Thongpraditchote S, Sithisarn P, Gritsanapan W (2013). Total phenolics and total flavonoids contents and hypnotic effect in mice of *Ziziphus mauritiana* Lam. Seed extract. Evid Based Complement Altern Med.

[CR50] Saslis-Lagoudakis CH, Hawkins JA, Greenhill SJ (2014). The evolution of traditional knowledge: environment shapes medicinal plant use in Nepal. Proc Biol Sci.

[CR51] Sawada LA, Monteiro VSDC, Rabelo GR (2014). *Libidibia ferrea* mature seeds promote antinociceptive effect by peripheral and central pathway: possible involvement of opioid and cholinergic receptors. Biomed Res Int.

[CR52] Shrestha S, Park J-H, Lee D-Y (2012). *Rhus parviflora* and its biflavonoid constituent, rhusflavone, induce sleep through the positive allosteric modulation of GABA(A)-benzodiazepine receptors. J Ethnopharmacol.

[CR53] Suksamrarn S, Suwannapoch N, Aunchai N (2005). Ziziphine N, O, P and Q, new antiplasmodial cyclopeptide alkaloids from *Ziziphus oenoplia* var. brunoniana. Tetrahedron.

[CR54] Tomazetti J, Avila DS, Ferreira APO (2005). Baker yeast-induced fever in young rats: characterization and validation of an animal model for antipyretics screening. J Neurosci Methods.

[CR55] Upadhyay B, Singh KP, Kumar A (2011). Ethno-veterinary uses and informants consensus factor of medicinal plants of Sariska region, Rajasthan, India. J Ethnopharmacol.

[CR56] Wahida B, Abderrahman B, Nabil C (2007). Antiulcerogenic activity of *Zizyphus lotus* (L.) extracts. J Ethnopharmacol.

[CR57] Walsh RN, Cummins RA (1976). The open-field test: a critical review. Psychol Bull.

[CR58] Ya’u J J, Abdulmalik UN, Yaro AH (2011). Behavioral properties of *Balanites aegyptiaca* in rodents. J Ethnopharmacol.

[CR59] Yeung W-F, Chung K-F, Poon MM-K (2012). Chinese herbal medicine for insomnia: a systematic review of randomized controlled trials. Sleep Med Rev.

[CR60] Ying C, Ning W, Ying L (2014). Anti-nociceptive and anti-inflammatory activities of the extracts of *Stauntonia chinensis*. Pak J Pharm Sci.

[CR61] Yu L, Jiang BP, Luo D (2012). Bioactive components in the fruits of *Ziziphus jujuba* Mill. against the inflammatory irritant action of Euphorbia plants. Phytomedicine.

[CR62] Yue Y, Wu S, Zhang H (2014). Characterization and hepatoprotective effect of polysaccharides from *Ziziphus jujuba* Mill. var. spinosa (Bunge) Hu ex H. F. Chou sarcocarp. Food Chem Toxicol.

[CR63] Zhao J, Jian J, Liu G (2014). Rapid SNP discovery and a RAD-based high-density linkage map in jujube (Ziziphus Mill.). PLoS One.

